# Direct cancer–stromal interaction increases fibroblast proliferation and enhances invasive properties of scirrhous-type gastric carcinoma cells

**DOI:** 10.1038/sj.bjc.6605309

**Published:** 2009-09-22

**Authors:** S Semba, Y Kodama, K Ohnuma, E Mizuuchi, R Masuda, M Yashiro, K Hirakawa, H Yokozaki

**Affiliations:** 1 Department of Pathology, Division of Pathology, Kobe University Graduate School of Medicine, Kobe, Japan; 2 Department of Surgical Oncology, Osaka City University Graduate School of Medicine, Osaka, Japan

**Keywords:** scirrhous-type gastric carcinoma, gastric fibroblast, cDNA microarray, VCAM-1, integrin-*α*4, epithelial–mesenchymal transition, matrix metalloproteinase

## Abstract

**Background:**

Scirrhous-type gastric carcinoma (SGC) exhibits an extensive submucosal fibrosis and extremely poor patient prognosis. We investigated the importance of the cancer–stromal interaction in the histogenesis of SGC.

**Methods:**

Gastric fibroblasts NF-25 and intestinal fibroblasts NF-j2 were co-cultured with SGC-derived (HSC-39) or non-SGC-derived (HSC-57 and HSC-64) cells. To identify genes that are up- or downregulated in NF-25, complementary DNA (cDNA) microarray analysis was performed. The antibody against vascular-cell adhesion molecule-1 (VCAM-1) was used for cell growth test and immunohistochemistry. Moreover, the impact of interaction with NF-25 fibroblasts on HSC-39 cells was investigated using western blot and reverse transcription-polymerase chain reaction.

**Results:**

HSC-39 cells stimulated growth of NF-25 but not NF-j2 when co-cultured. Induction of VCAM-1 in NF-25 fibroblasts was identified, which was specific when co-cultured with HSC-39 but not with non-SGC-derived HSC-57 and HSC-64 cells. Neutralising antibody to VCAM-1 suppressed NF-25 growth in dose-dependent manners. In tissue samples, positive immunoreactivity of VCAM-1 in SGC-derived fibroblasts was significantly higher than that in non-SGC-derived fibroblasts. Furthermore, interaction with NF-25 fibroblasts not only induced the epithelial–mesenchymal transition-like change, but also expressions of matrix metalloproteinase- related genes in HSC-39 cells.

**Conclusion:**

Direct interaction between SGC cells and gastric fibroblasts establishes the tumour microenvironment and reinforces the aggressiveness of SGC.

Scirrhous-type gastric carcinoma (SGC), also called diffuse-type gastric carcinoma, or linitis plastica, is characterised by diffuse infiltration and rapid proliferation of cancer cells accompanied by extensive submucosal fibrosis and resultant fibrous thickening of the wall (Otsuji *et al*, 2004). The prognosis of patients with SGC is extremely poor, mostly due to frequent incidence of lymph node metastasis and peritoneal dissemination of cancer cells; therefore, understanding the molecular mechanism underlying these characteristics of SGC for the development of new therapies has become urgent (Ikeguchi *et al*, 2004; Nakamura *et al*, 2006).

In cancer, stromal change at the invasive front is recognised as a ‘desmoplastic response’, which can provide a significant impact on the proliferation and invasiveness of cancer cells (De Wever and Mareel, 2003). The cancer-associated fibroblasts (CAFs), which consist of fibroblasts and myofibroblasts, build a cancer–stromal interaction by two distinct mechanisms, the efferent pathway and the afferent pathway (Hwang *et al*, 2008). In the efferent pathway, various autocrine and paracrine mediators have been found, including growth factors, cytokines and interleukins. In particular, transforming growth factor-*β* strongly promotes not only chemotaxis of CAFs (Postlethwaite *et al*, 1987), but also conversion of non-invasive lesions to invasive ones (Cui *et al*, 1996). In addition, several factors involving keratinocyte growth factor (Nakazawa *et al*, 2003), hepatocyte growth factor (Tendo *et al*, 2005) and interleukin-1*β* (Yashiro *et al*, 2007) have been documented as important mediators activating paracrine or autocrine signalling between SGC cells and CAFs, all of which result in the promotion of tumour growth and progression. In the afferent pathway, in turn, direct interaction between cancer cells and CAFs triggers various intracellular signalling pathways; for instance, the cell adhesion molecule N-cadherin is thought to play an important role in the regulation of many intracellular responses that activate cancer cell motility and invasiveness (Hazan *et al*, 2000). Thus, the microenvironment for acceleration of cancer cell invasion and progression is intricately composed with interactions with CAFs. Moreover, the significance of organ-specific fibroblasts in the proliferation and invasion of breast cancer cells has been suggested (Yashiro *et al*, 2005); however, little is known about the role of gastric fibroblasts as CAFs during the progression of SGC.

In general, the epithelial–mesenchymal transition (EMT) and degradation of the extracellular matrix (ECM) are thought to be early events during multistep tumour invasion and metastasis. The EMT is a morphogenic process in which cells lose their epithelial characteristics such as cell polarity and cell–cell contact, and gain mesenchymal properties such as increased motility (Berx *et al*, 2007). Although EMT has been originally described in its functions during embryogenic development (Duband *et al*, 1995; Shook and Keller, 2003), accumulating evidence has shown that it plays a critical role in tumour invasion and metastasis, particularly in the process of the detachment and migration of cancer cells from the primary tumour and establishment of metastatic sites in distant organs (Thompson *et al*, 2005). The EMT of cancer cells is often induced by various transcription factors such as Snail, Twist and Slug (Lombaerts *et al*, 2006), resulting in downregulation of epithelial markers such as E-cadherin and upregulation of mesenchymal markers such as vimentin. In SGC, retained expression of the mesenchymal-like genes induced by hedgehog transcription factor has been reported (Ohta *et al*, 2009). On the other hand, the balance between local production levels of matrix metalloproteinases (MMPs) and tissue inhibitors of metalloproteinases determines the capability of degradation of ECM. Elevated levels of MMP expression have been documented in various human malignancies (Liotta *et al*, 1991), and overexpression of MMP2 and is closely correlated with high incidence of invasion and metastasis (Nomura *et al*, 1996), which is activated by membrane type-1-MMP (MT1-MMP) (Sato *et al*, 1994). Hence, diversified experimental approaches are necessary to understand the biological, histopathological and clinical properties of SGC.

In this study, we attempted to identify the novel specific factors that may be altered in gastric fibroblasts when co-incubated with SGC-derived cells. Genes differentially expressed in gastric fibroblasts in the presence or absence of direct co-culture with SGC cells were investigated using complementary DNA (cDNA) microarray analysis. Furthermore, the effects of cell–cell contact on the invasive properties of SGC cells were also assessed to understand the biological significance of the cancer–stromal interaction in the histogenesis of SGC.

## Materials and methods

### Cell lines and cell growth test

Human SGC cell line HSC-39 (passage 20) and non-SGC cell lines HSC-57 (passage 10) and HSC-64 (passage 10) were provided by Dr Yanagihara (National Cancer Institute Research Center, Tokyo, Japan). HSC-39 cells (clonal) were derived from the peritoneal ascites of a 54-year-old male patient with SGC (signet-ring cell carcinoma; Yanagihara *et al*, 1991). HSC-57 (clonal) and HSC-64 (clonal) cells were derived from the ascites of patients with well- and poorly differentiated-type adenocarcinomas of the stomach, respectively (unpublished data). NF-25 fibroblasts (heterogenous, passage 4) were established from a 77-year-old male patient with early gastric carcinoma who had a distal gastrectomy. Gastric fibroblasts from the non- tumour al wall were cultured and isolated. NF-j2 fibroblasts (heterogenous, passage 4) were also isolated from the jejunum of a male patient with pancreatic cancer. The patient had a pancreaticoduodenuctomy and fibroblasts were obtained from the cancer-free jejunal wall. Cells were routinely maintained in RPMI-1640 supplemented with 10% foetal bovine serum. NF-25 and NF-j2 fibroblasts seeded at a density of 1.0 × 10^5^ cells well^−1^ in six-well plates were incubated in the presence or absence of direct co-incubation of the same number of HSC-39 cells. For indirect incubation with HSC-39 cells, a 1-*μ*m pore-size Boyden Chamber (BD Falcon, Franklin Lakes, NJ, USA) was used. We counted the number of the cells with cell counting chamber. The Dynabeads Epithelial Enrich system (Dynal Biotech ASA, Oslo, Norway) was used to separate HSC-39 cells from co-incubated NF-25 and NF-j2 fibroblasts. The numbers of NF-25 fibroblasts were counted in the presence or absence of neutralising antibodies to vascular-cell adhesion molecule-1 (VCAM-1) (Santa Cruz, Santa Cruz, CA, USA) and integrin-*α*4 (Santa Cruz).

### Immunofluorescence

Twenty-four hours after co-culture, HSC-39 cells and NF-25 fibroblasts in chamber slides were fixed with 4% formalin and incubated with blocking solution containing 1% bovine serum albumin. Cells were incubated with antibodies against vimentin (1 : 1000 dilution; Dako, Glustrup, Denmark), cytokeratin (1 : 1000 dilution; Dako) and VCAM-1. After washing, the slides were incubated with the secondary antibodies, a mixture of Cy2-conjugated anti-mouse and Cy3-conjugated anti-rabbit IgG antibodies (1 : 10 000 dilution).

### cDNA microarray analysis

Total RNA was extracted from NF-25 fibroblasts (single culture and co-cultured with HSC-39) using the RNeasy kit (Qiagen, Hilden, Germany). A cDNA microarray was prepared by IntelliGene HS Human Expression CHIP (Takara Bio, Otsu, Japan), containing probes for 16 600 characterised human genes and expressed sequence tags. *In vitro* transcription, oligonucleotide array hybridisation and scanning were performed according to Takara Bio instructions. Briefly, double-stranded cDNA was synthesised from total RNA and was labelled with the RNA Fluorescence Labeling Core kit (Takara Bio). Arrays were then scanned with the GeneArray scanner (Agilent Technologies, Palo Alto, CA, USA) to obtain image and signal intensities. After data normalisation, significance analysis of microarray (SAM) plot analysis was performed and significantly altered genes were identified in accordance to the manufacture's instructions (http://chem.agilent.com).

### Immunohistochemistry

A total of 37 formalin-fixed and paraffin-embedded specimens of sporadic SGCs and non-SGCs surgically removed at Kobe University Hospital (Kobe, Japan) were used. None of these cases received adjuvant chemotherapy or radiotherapy before surgery. Informed consent was obtained from all patients and the study was approved by the Kobe University Institutional Review Board. Histological examination was performed according to Japanese Classification of Gastric Carcinoma (Japanese Gastric Cancer Association, 1999). A modified version of the immunoglobulin enzyme bridge technique with the LSAB kit (Dako) was used. Briefly, deparaffinised and rehydrated 4-*μ*m sections were autoclaved to retrieve antigenicity. After blocking endogenous peroxidase and non-specific binding sites, antibodies against VCAM-1(1 : 200 dilution), E-cadherin (1 : 200 dilution; Dako) and Snail (1 : 100 dilution; Abcam, Cambrige, UK) were applied to the sections. Sections were then incubated with biotinylated goat anti-mouse or anti-rabbit IgG (1 : 10 000 dilution) and streptavidin conjugated to horseradish peroxidase (HRP). Chromogenic fixation was carried out by immersing the sections in a solution of 3,3′-diaminobenzidine. Sections were counterstained with Mayer's haematoxylin. The degrees of immunoreactivities of each molecule were graded according to the number of stained cells and the staining intensity in individual cells: (–), almost no positive cells or <30% of tumour cells showing weak immunoreactivity and (+), >30% of tumour cells showing weak immunoreactivity or tumour cells showing intense immunoreactivity.

### Western blotting

The cells were lysed in a buffer containing 50 mM Tris-HCl (pH 7.4), 125 mM NaCl, 0.1% Triton X-100 and 5 mM ethylenediaminetetraacetic acid containing 1% protease inhibitor cocktail (Sigma, St Louis, MO, USA). Proteins (20 *μ*g) were separated by sodium dodecylsulphate–polyacrylamide gel electrophoresis followed by electrotransfer onto Hybond C membrane (Millipore, Bedford, MA, USA). After blotting with antibodies against VCAM-1 (1 : 1000 dilution), integrin-*α*4 (1 : 1000 dilution), FAK (1 : 1000 dilution; Santa Cruz), paxillin (1 : 1000 dilution; BD Transduction, Lexington, KY, USA), E-cadherin (1 : 1000 dilution), vimentin (1 : 1000 dilution), Snail (1 : 500 dilution) and *β*-actin (1 : 10000 dilution; Sigma), HRP-conjugated anti-mouse or anti-rabbit IgGs (1 : 10000 dilution, GE Healthcare, Little Chalfont Buckinghamshire, UK) were used as secondary antibody. The signals were visualised with enhanced chemiluminescence.

### RT–PCR

Reverse transcription-polymerase chain reaction (RT–PCR) was performed with a OneStep RT–PCR assay kit (Qiagen). The primer sets used in the present study were shown in Table 1. Each 25-*μ*l reaction mixture containing 10 ng of total RNA was amplified for 30 cycles with the following regimen: reverse transcription at 50°C for 30 min; denaturation at 94°C for 30 s; annealing at 58°C for 30 s and extension at 72°C for 1 min. The products were underwent electrophoresis on 2% agarose gel.

## Results

### The effects of co-incubation with SGC cells on proliferation of fibroblasts

**Figure 1:**
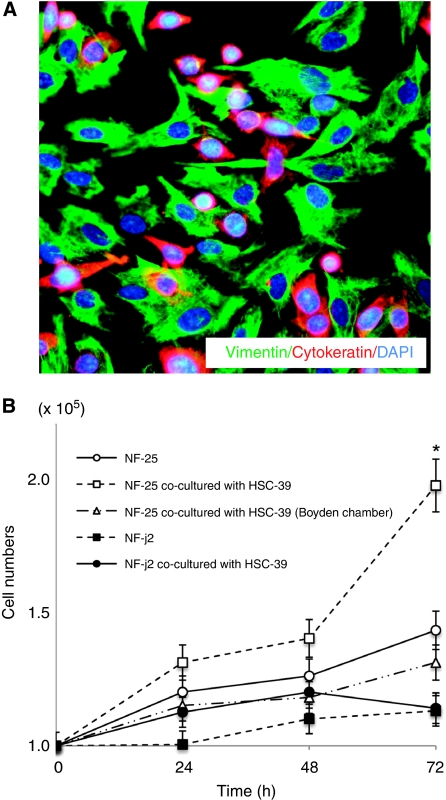
Cell–cell contact with SGC-derived HSC-39 cells upregulated NF-25 gastric fibroblasts’ growth. (**A**) Immunofluorescence of NF-25 fibroblasts co-cultured with HSC-39 cells. NF-25 fibroblasts and HSC-39 cells were stained with vimentin (green) and cytokeratin (red) ( × 200). (**B**) Growth curves of NF-25 gastric fibroblasts and NF-j2 intestine fibroblasts in the presence or absence of co-incubation with HSC-39 cells. Before counting of the numbers of NF-25 and NF-j2 fibroblasts, HSC-39 cells co-cultured were excluded by separation by a magnetic beads method. To examine the effect of soluble factors, NF-25 fibroblasts and HSC-39 cells were separately co-maintained using a 1-*μ*m pore-sized Boyden Chamber inserts. ^*^<0.01.

To investigate the effect of SGC cells on the proliferative activity of gastric fibroblasts, the numbers of NF-25 gastric fibroblasts cultured in the presence or absence of SGC-derived HSC-39 cells were counted. HSC-39 cells were originally established from ascites of a patient with SGC and the cells, therefore, do not show typical shape of adherent cells but form colonies in the absence of NF-25 fibroblasts; nevertheless, the HSC-39 cells exhibited direct attachment and assembly with the NF-25 fibroblasts at the bottom of the culture dish, although no morphological change was observed in these lines (Figure 1A). In addition, the number of NF-25 fibroblasts was dramatically elevated when the cells were co-cultured with HSC-39 cells (*P*<0.001; Figure 1B). To examine the possible effect of soluble factors in the culture media, NF-25 fibroblasts and HSC-39 cells were separately co-maintained using a 1-*μ*m pore-sized Boyden Chamber inserts; however, no significant increase of cell proliferation was detected (Figure 1B).

Yanagihara *et al* (2004) previously reported that orthotopic implantation of HSC-44PE SGC cells caused a xenograft to develop in the stomach, showing extensive fibrosis with only sparse tumour cell infiltration; however, such proliferation of fibroblasts was not observed in metastatic sites including the skin, lymph node and lung, suggesting that the phenomenon is orthotopic. Thus, we next evaluated the proliferative activity of NF-j2 intestinal fibroblasts to examine whether the effect of co-culture with HSC-39 cells is tissue specific or not. HSC-39 cells did not show cell–cell contact with NF-j2 fibroblasts when co-cultured and floated above the NF-j2 fibroblasts. There was no induction of cell growth of NF-j2 fibroblasts (Figure 1B).

### Upregulation of VCAM-1 expression is specifically induced by SGC cells in gastric fibroblasts

**Figure 2:**
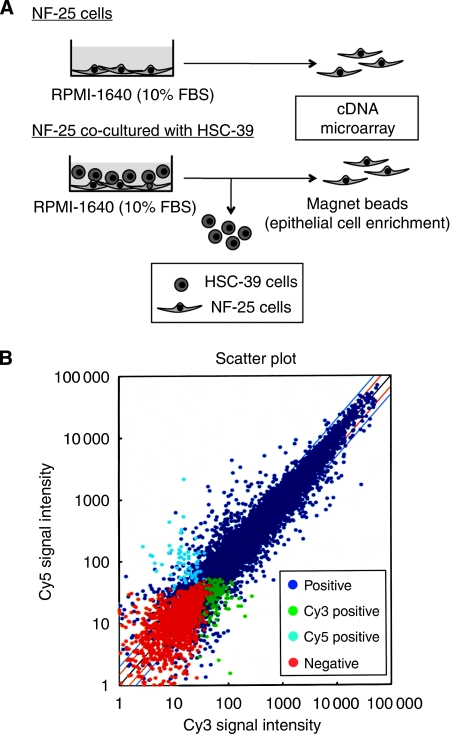
Identification of genes differentially expressed in NF-25 gastric fibroblasts in the presence or absence of cell–cell contact with HSC-39 cells. (**A**) Illustration of the strategy of cDNA microarray. NF-25 fibroblasts (1 × 10^5^ cells dish^−1^) were maintained for 48 h in the presence or absence of HSC-39 cells (1 × 10^5^ cells dish^−1^). HSC-39 cells were separated with magnetic beads epithelial cell enrichment. (**B**) Results of SAM plot analysis. Cy5 positive (light blue), genes upregulated in NF-25 fibroblasts co-cultured with HSC-39 cells; Cy3 positive (green), genes upregulated in NF-25 fibroblasts; positive (blue), genes equally expressed and negative (red), genes, which were not expressed.

To identify the molecules specifically up- and downregulated in NF-25 fibroblasts, we performed a cDNA microarray analysis using total RNAs from NF-25 fibroblasts cultured in the presence or absence of HSC-39 cells (Figure 2A). The change in the gene expression profile of the NF-25 fibroblasts with co-incubation with HSC-39 cells compared with the NF-25 fibroblasts without co-culture with HSC-39 cells did not involve a large number of genes: after normalisation and revision of raw data, 233 genes (>2.5-fold upregulated, 107 genes and <0.4-fold downregulated, 126 genes) were identified as showing statistically significant differences (Figure 2B). The accuracy of the microarray analysis was confirmed by real-time RT–PCR analysis of the expression of six randomly selected differentially expressed genes, as the results showed good concordance with the microarray data in terms of fold change of gene expression (data not shown). Among the 13 adhesion-related genes that were affected (upregulated, eight genes and downregulated, five genes; Table 2), we finally decided to focus on VCAM-1 gene transcript (GDB accession no. NM_001078.2), which showed 4.60-fold upregulation in NF-25 fibroblasts co-cultured with HSC-39 cells.

**Figure 3:**
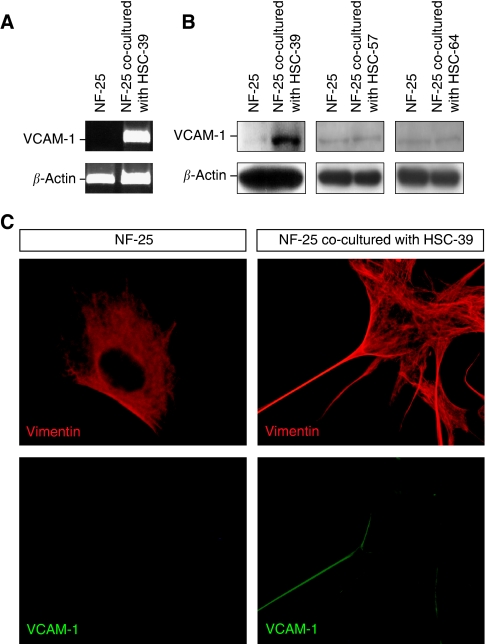
Induction of VCAM-1 expression in NF-25 gastric fibroblasts is specifically induced by direct interaction with HSC-39 cells. NF-25 fibroblasts were co-incubated with HSC-39 (SGC-derived), HSC-57 (non-SGC-derived) and HSC-64 (non-SGC-derived) cells for 48 h. Cancer cells were separated with magnetic beads epithelial enrichment. (**A**) Results of RT–PCR analysis. The levels of *β-actin* expression were used as control. (**B**) Results of western blotting. The levels of *β*-*actin* expression were used as control. (**C**) Immunofluorescence and morphological changes of NF-25 fibroblasts in the presence or absence of co-culture with HSC-39 cells ( × 200). NF-25 fibroblasts were visualised with antibodies against vimentin (red) and VCAM-1 (green).

We next confirmed whether VCAM-1 induction in NF-25 fibroblasts is a SGC-specific event or can be seen with non-SGC cells. Induction of VCAM-1 at both the mRNA and protein levels was dramatically observed when the NF-25 fibroblasts were co-incubated with HSC-39 cells (Figure 3A and B). In NF-25 fibroblasts VCAM-1 protein expressed at the cellular membrane with a distinct spindle morphology (Figure 3C). However, no induction was found in the levels of VCAM-1 expression in NF-25 fibroblasts when co-cultured with HSC-57 and HSC-64 cells, both of which were established from patients with non-SGC.

### The significance of the integrin-*α*4–VCAM-1 signalling pathway on gastric fibroblast proliferation

**Figure 4:**
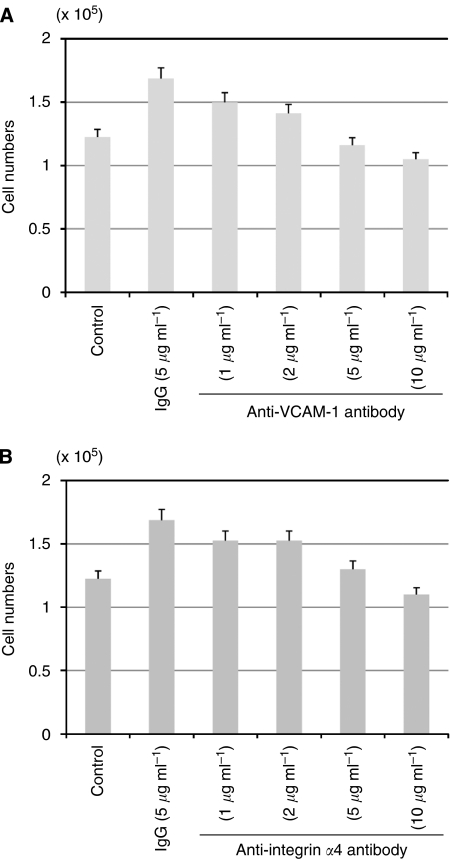
Effects of neutralising antibodies to VCAM-1 and integrin-*α*4 on NF-25 fibroblasts growth in the presence of co-culture with HSC-39 cells. NF-25 fibroblasts (1 × 10^5^ cells dish^−1^) were co-maintained with HSC-39 cells (1 × 10^5^ cells dish^−1^) and the various amounts of each antibody were added to the media. The numbers of NF-25 fibroblasts were counted 48 h after addition of these neutralising antibodies. (**A**) Effects of anti-VCAM-1 antibody (0–10 *μ*g ml^−1^). (**B**) Effects of anti-integrin-*α*4 antibody (0–10 *μ*g ml^−1^). Non-specific mouse IgG (5 *μ*g ml^−1^) was used as negative control. The experiments were performed thrice.

The effect of the neutralising antibody to VCAM-1 was investigated to assess whether induction of VCAM-1 expression by co-culture with HSC-39 cells can promote the growth activity of NF-25 cells. Addition of anti-VCAM-1 antibody to the culture media effectively suppressed the growth-promoting effect of cell–cell contact with HSC-39 in a dose-dependent manner (Figure 4A). Adhesion molecule integrin-*α*4 is able to bind with VCAM-1, and subsequently activates the intracellular signalling that promotes detachment of cell–cell adhesion and increased cell migration activity (Bogetto *et al*, 2000; Klemke *et al*, 2007). Similarly, the number of NF-25 fibroblasts was decreased when the cells were co-cultured with HSC-39 cells in media containing anti-integrin-*α*4 antibody in a dose-dependent manner (Figure 4B).

**Figure 5:**
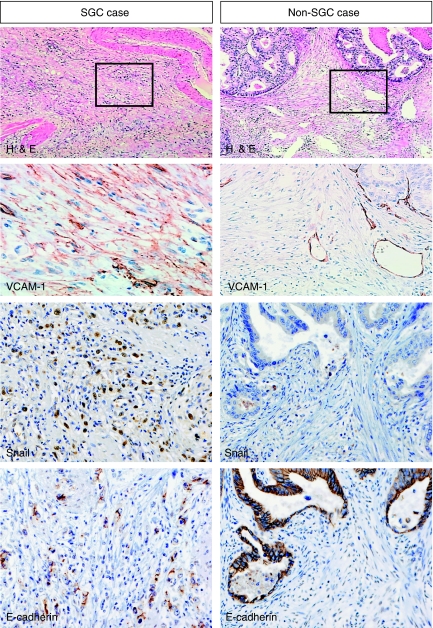
Immunohistochemistry of VCAM-1, Snail and E-cadherin expressions under VCAM-1-positive SGC (signet-ring cell carcinoma) condition and VCAM-1-negative non-SGC condition (moderately differentiated tubular adenocarcinoma). Histological examination was performed by haematoxylin and eosin (H&E) staining. Original maginification: × 200.

According to these results obtained from the *in vitro* experiments, we investigated VCAM-1 expression in sporadic SGC and non-SGC cases. The results of immunohistochemistry are summarised in Table 3, and representative illustrations are shown in Figure 5. Overall, VCAM-1 expression in CAFs was detected in 14 (38%) of 37 SGC and non-SGC cases examined. Expression of VCAM-1 was detected in 11 (61%) of 18 SGC cases in which extensive growths of CAFs surrounded the cancer cells, and three (15%) of 17 non-SGC cases in which cancer cells had a tubular formation with desmoplastic change in CAFs. The frequencies of VCAM-1 expression in CAFs were significantly different in SGC cases and non-SGC cases (*P*=0.004).

### Cell–cell contact with gastric fibroblasts promotes EMT-like change and induces MMP production in SGC cells

**Figure 6:**
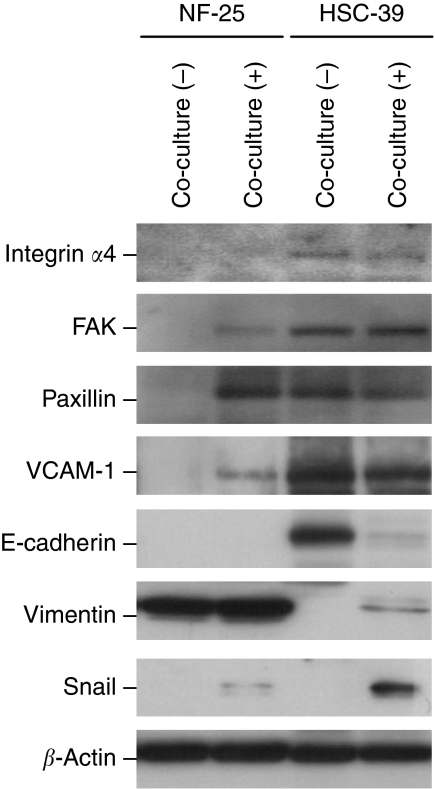
Effects of cell–cell contact on downregulation of cell adhesion molecules in NF-25 fibroblasts and induction of EMT-like change in HSC-39 cells. Results of western blot. Twenty-four hours after incubation of NF-25 and HSC-39 cells, the cells were separated by magnetic beads method. The levels of *β*-actin expression were used as control.

Altered characteristics of HSC-39 cells by co-incubation with NF-25 fibroblasts were analysed. The protein levels of FAK and paxillin, the VCAM-1-integrin-*α*4 signalling pathway-related molecules (Liu and Ginsberg, 2000; Liu *et al*, 2002), were upregulated in NF-25 fibroblasts when co-cultured with HSC-39 cells; however, there was no significant change in the integrin-*α*4 expression levels (Figure 6). On the other hand, in HSC-39 cells no alteration was found in the levels of these proteins involved in the integrin-*α*4–VCAM-1 signalling pathway. Interestingly, induction of Snail, an EMT-related transcription factor, was detected, accompanied by decreased levels of E-cadherin expression and increased levels of vimentin expression (Figure 6). Indeed, intense immunoreactivity of Snail was detected in VCAM-1-positive SGC cases that were accompanied by low E-cadherin expression (Figure 5).

**Figure 7:**
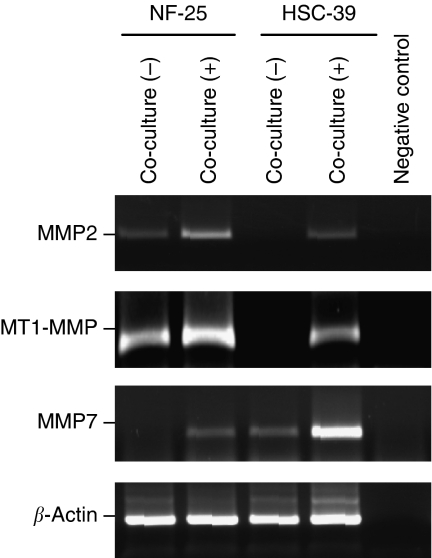
Effects of cell–cell contact on upregulation of MMPs in HSC-39 cells. Results of RT–PCR. Twenty-four hours after incubation of NF-25 fibroblasts and HSC-39 cells, the cells were separated by magnetic beads method. The levels of *β-actin* expression were used as control.

Similarly, we further investigated altered expressions of MMPs in HSC-39 cells, which function for the degradation of ECM and allow cancer cells to invade into tissues (Sato *et al*, 1994). The mRNA levels of *MMP-2*, *MT1-MMP* and *MMP7* in HSC-39 cells were found to be significantly increased (Figure 7).

## Discussion

In this study, we assessed the significance of co-culturing NF-25 gastric fibroblasts and SGC-derived HSC-39 cells as a clue for elucidating the pathogenesis of SGC, typically accompanied by extraordinary thickening of the gastric wall, clinically rapid progression and resultant poor prognosis of patients. Supporting these properties of SGC, co-incubation with SGC-derived cells strongly enhanced the growth of gastric fibroblasts and, in turn, influenced the invasive/metastatic potential in SGC cells themselves. In addition, we found that cell–cell contact-mediated induction of VCAM-1 in NF-25 cells increased its proliferation activity, and neutralising antibodies to VCAM-1 and integrin-*α*4 decreased cell growth in a dose-dependent manner, respectively. This is the first report to demonstrate the possible role of activation of the VCAM-1-integrin-*α*4 signalling pathway for the promotion of gastric fibroblast proliferation. Previous studies have shown that integrin-*α*4 expressed in leukocytes functions as a receptor of VCAM-1, which is expressed in endothelial cells and ECM fibronectin (Chan and Aruffo, 1993; Postigo *et al*, 1993); therefore, VCAM-1 is believed to have a crucial role in supporting the capture and immobilisation of leukocytes in the bloodstream as a scaffold. Nevertheless, we detected expression of VCAM-1 in a large fraction of SGC cases, confirming mediation of VCAM-1 in the development of SGC. In human T-lymphoblastic lymphoma, VCAM-1 is expressed not only in lymphoma cells, but also on both the apical and the basolateral surfaces of endothelial cells, consequently activating the sequential transmigration and intravasation of lymphoma cells (Bogetto *et al*, 2000). Similarly, the high-affinity interaction between integrin-*α*4 and VCAM-1 promoted trans-endothelial migration in melanoma cells (Klemke *et al*, 2007). Abundant VCAM-1 expression in CAFs may help promote the aggressiveness of SGC cells, and this phenomenon may contribute to the rapid spread and vascular infiltration of SGC cells *in vivo*. Preoperative serum concentrations of a soluble form of VCAM-1 in the sera of gastric cancer patients were significantly higher when compared with those of healthy controls; in addition, there were significant associations of elevated VCAM-1 levels with disease stage, gastric wall invasion, lymph node involvement and presence of distant metastases (Alexiou *et al*, 2002). Although the possible function of circulating VCAM-1 in the development of distant metastastic sites remains unknown, the findings suggest that evaluation of VCAM-1 levels in serum or biopsy specimens can be a novel marker for predicting a patient's risk of carcinoma metastasis and recurrence after surgery. Furthermore, VCAM-1 is expressed mainly from CAFs in gastric carcinoma tissues rather than cancer cells. Elevation of VCAM-1 levels on the cell surface of gastric fibroblasts and its secretion to the stroma may have a great impact on the proliferation and migration of SGC cells.

Recent studies have documented that CAFs are implicated in important aspects of epithelial solid-tumour biology, such as cancer progression, tumour growth, angiogenesis and metastasis by sustained expression of stromal-derived factor-1 (SDF-1), also known CXCL12 (Yang *et al*, 2008). Indeed, SDF-1 secretion by bone morphogenetic protein-2 increased tubular formation of microvascular endothelial cells and recruiting endothelial progenitor cells (Orimo *et al*, 2005), and stimulated tumour growth directly, acting through CXCR4, which is expressed by carcinoma cells. Thus, SDF-1 is thought to play a central role in establishing the niche for cancer progression and metastasis. Nevertheless, in the present study no soluble factor was suggested to promote NF-25 fibroblast growth when separately co-cultured with HSC-39 cells, and increased mRNA levels of *SDF-1* expression were not detected in NF-25 fibroblasts co-cultured with HSC-39 cells by cDNA microarray analysis (data not shown). Rather, we found that proliferation of NF-25 fibroblasts was accelerated by direct co-culture with HSC-39 cells, with significant induction of VCAM-1 expression. In addition, this VCAM-1 induction was detected only in NF-25 gastric fibroblasts and not in NF-j2 intestinal fibroblasts, which was a specific effect caused by interaction with SGC-derived cells. These findings suggest the importance of direct interaction between SGC and gastric fibroblasts for the construction of a niche that is capable of promoting fibrosis of the gastric wall and increasing the malignant behaviour of cancer cells. In this study, we did not thoroughly exclude the possible mediation of soluble factors that were secreted from both HSC-39 cells and NF-25 fibroblasts by direct co-culture. Further investigation will be required to clarify the participation of such extracellular stimuli during the progression and establishment of distant metastasis in patients with SGC.

Many reports have investigated the impact of the cancer–stromal interaction in human malignancies, and have shown the importance of cell–cell contact for the establishment of a tumour microenvironment that may affect cancer cell motility and invasiveness, EMT, angiogenesis and distant metastasis (De Wever and Mareel, 2003). Certainly, EMT in clinical cancer specimens is defined as loss of junctional E-cadherin; switch to other cadherins (e.g., N-cadherin); degradation of cell–cell adhesion; apicobasal polarity and tissue architecture; pleiotropic cell shape; nuclear *β-catenin*, *Snail* or *Slug* expression; and otherwise unexpected expression of mesenchymal markers such as vimentin (Thompson *et al*, 2005). However, we do not use the term ‘EMT’ for the biological phenomena observed in this study because disorderly differentiation, loss of cell polarity and loss of lineage-specific or tissue-specific cytologic features are defining aspects of carcinomas except ‘so-called’ carcinosarcoma. Tarin (2005) recommended not to use the word ‘EMT’ in cancer cells, particularly when expressing scattered single-cell infiltration by diffuse (or signet-ring cell) carcinoma of the stomach. Therefore, we expressed this reaction of HSC-39 cells as ‘EMT-like change’ in SGC cells. According to our experiment using co-culture of NF-25 fibroblasts and HSC-39 cells, direct interaction with gastric fibroblasts induced Snail expression, and resultant E-cadherin suppression and vimentin induction in HSC-39 cells. We assumed the hypothesis that the levels of vimentin and E-cadherin expression in HSC-39 cells examined have been modified for some reason, which was consequently restored when the cells were co-cultured with NF-25 fibroblasts. However, HSC-39 cells originally express high levels of E-cadherin (Oyama *et al*, 1994), and we finally concluded that EMT-like change was induced in HSC-39 cells by contacting with NF-25 fibroblast. A previous study investigated differential gene expression profiles of SGC using a cDNA microarray and found that downregulation of E-cadherin and integrin-*β*4 expression in SGC-derived cell lines was associated with high potential of metastases to the peritoneum and lymph nodes (Hippo *et al*, 2001), also supporting that induction of EMT-like change by having cell–cell contact with NF-25 fibroblasts may promote cancer aggressiveness, which is probably mediated by the induction of Snail. A recent study by Ohta *et al* (2009) suggested that EMT may have a pivotal role in the progression and development of SGC; therefore, understanding the mechanism of this EMT-like change in SGC cells is necessary for the development of a novel chemotherapeutic approach against SGC in the future.

## Figures and Tables

**Table 1:** The primer sets for RT-PCR

**Gene**	**Primer sequences**	**Annealing temp. (°C)**	**Product size (bp)**
*VCAM-1*	F: 5-CATCCACAAAGCTGCAAGAA-3	58	529
	R: 5-GCCACCACTCATCTCGATTT-3		
*MMP2*	F: 5-GCGACAAGAAGTATGGCTTC-3	58	390
	R: 5-TGCCAAGGTCAATGTCAGGA-3		
*MT1-MMP2*	F: 5-GGCAACATAATGAAATCACTT-3	58	301
	R: 5-TCGGCAGAGTCAAAGTGGGT-3		
*MMP7*	F: 5-AAACTCCCGCGTCATAGAAAT-3	58	395
	R: 5-TCCCTAGACTGCTACCATCCG-3		
*β-Actin*	F: 5-GCACTCTTCCAGCCTTCCTTC-3	58	237
	R: 5-GGAGTACTTGCGCTCAGGAGG-3		

Abbreviations: *MMP*, matrix metalloproteinase; *MT1*-*MMP2*, membrane type-1-MMP-2; RT-PCR, reverse transcription-polymerase chain reaction; temp., temperature; *VCAM*, vascular-cell adhesion molecule.

**Table 2:** CAM-related genes differentially expressed in NF-25 gastric fibroblasts co-cultured with SGC-derived HSC-39 cells

**Accession number**	**Gene title**	**Symbol**	**Fold**
Upregulated genes (fold change >2)			
NM_001306.2	Claudin 3	*CLDN3*	6.61
NM_007183.1	Plakophillin	*PKP3*	5.53
NM_000228.1	Laminin, *β*-3	*LAMB3*	5.22
NM_001793.2	Cadherin 3, type-1 (P-cadherin)	*CDH3*	4.74
NM_000610.2	CD44 antigen	*CD44*	4.73
NM_001078.2	Vascular-cell adhesion molecule-1, transcript variant 1	*VCAM-1*	4.60
NM_001305.2	Claudin 4	*CLDN4*	4.27
NM_144503.1	F11 receptor, transcription variant 1	*F11R*	3.34
Downregulated genes (fold change <0.5)			
NM_000094.2	Collagen, type IV, *α*1	*COL7A1*	0.26
NM_002160.1	Tenascin C (hexabrachion)	*TNC*	0.28
NM_002203.2	Integrin-*α*2 (CD49B)	*ITGA2*	0.35
NM_001901.1	Connective tissue growth factor	*CTGF*	0.39
NM_014000.1	Vinclin, transcription variant meta-VCL	*VCL*	0.40

Abbreviations: CAM, cell adhesion molecule; HSC, human SGC cell line; SGC, scirrhous-type gastric carcinoma.

**Table 3:** Results of immunohistochemistry of VCAM-1 expression in CAFs in SGC and non-SGC tissues

	**Total**	**VCAM-1(+)**	**(%)**	**VCAM-1(–)**	**(%)**	***P*-value** ^*^
Total	37	14	(38)	23	(62)	
SGC cases	18	11	(61)	7	(39)	0.004
Non-SGC cases	19	3	(15)	16	(85)	

Abbreviations: CAF, cancer-associated fibroblast; SGC, scirrhous-type gastric carcinoma; VCAM, vascular-cell adhesion molecule.

^*^P-values less than 0.05 were considered statistically significant.
